# Virtual Reality Application for Teaching Complex Congenital Heart Defect Anatomy: Design and Development Study

**DOI:** 10.2196/74429

**Published:** 2025-12-22

**Authors:** Kevin Muneton, David Buyck, Carlos-Eduardo Guerrero-Chalela, Shanti Narasimhan, Paul A Iaizzo

**Affiliations:** 1 The Visible Heart Laboratories, University of Minnesota, B172 Mayo, MMC195, 420 Delaware St SE, Minneapolis, MN, 55455, United States, 1 5618713359; 2 Department of Bioinformatics and Computational Biology, University of Minnesota, Minneapolis, MN, United States; 3 3D Printing and Modelling Centre La Cardio-Andes, Institute of Cardiology, Fundacion Cardioinfantil Instituto de Cardiología, Bogotá, Colombia; 4 Division of Pediatric Cardiology, University of Minnesota, Minneapolis, MN, United States

**Keywords:** medical education, virtual reality, congenital heart disease, congenital heart defect, anatomy, innovation

## Abstract

**Background:**

Medical education still faces challenges in teaching complex human cardiac anatomy to a wide range of learners, especially in the subject of congenital heart defects. Traditional educational methods such as cadaver dissection and the use of textbooks still face some limitations, for example, specimen availability and student comfort. The objective of this research is to use newer technologies, like virtual reality (VR), to teach the anatomy of congenital heart defects (CHDs) of 3D heart models from real computed tomography (CT)-scan images, to offer a more profound learning experience for learners and mentors.

**Objective:**

The objective of this study is to propose and implement an innovative pipeline for a VR-based educational application for CHDs, and to assess its acceptance and suitability for medical education by evaluating potential future users.

**Methods:**

Heart images of anonymized CT scans were used to create high-fidelity 3D cardiac models, using Materialise Mimics Core and 3-matic Medical. These models were next integrated into a Unity-powered VR application. A heterogeneous group of cardiologists, biomedical engineers, and medical trainees visiting the Visible Heart Laboratories was invited to this pilot study to assess perceived effectiveness and satisfaction of this VR app for CHD education.

**Results:**

A total of 9 participants were included in the study, comprising 4 (44%) cardiologists, 2 (22%) biomedical engineers, and 2 (22%) medical trainees. Mean perception based on potential for CHD education of this VR-app (4.56, SD 0.53), was higher than traditional methods (3.22, SD 0.026*; P*=.008). Participants rated this application more suitable for medical students (mean=3.22, SD 0.83; *P*=.02) and patient education (mean=4.11, SD 1.05; *P*=.03).

**Conclusions:**

Our developed VR application offers an innovative approach to teaching the complex cardiac anatomy of CHDs using models from CT scans. This research shows how newer technologies can be optimized for medical education with the advantage of 3D visualization and a teaching environment without geographical limitations. Future work should focus on Extended Reality (XR) integration, adding more heart and organ models, as well as performing long-term efficacy studies.

## Introduction

Current curricula in medical education often face the challenges of keeping pace with newly developed teaching methods: for example, these include those introduced by technological advancements such as the use of web applications, virtual reality (VR), and extended reality (XR) [1]. The study of complexities and variations within human anatomy has historically been based on the use of fresh cadavers, hearts preserved in formaldehyde, and the study of anatomy textbooks [2,3]. The use of new technologies in medical education, such as XR and VR, is particularly valuable given the complex spatial architecture of the human heart [4,5]. This complexity is further amplified in the context of teaching congenital heart defects (CHD), where anatomical variations are highly diverse. Students face significant cognitive challenges in understanding these conditions due to the intertwined vascular structures, dynamic relationships between cardiac components, and the wide variability of defects that can occur from one patient to another [5,6].

The use of traditional dissection and the study of macroscopic anatomy, as well as generating human models, while of particular importance in acquiring practical skills that are difficult to replace [7-9], are methods with numerous limitations. Today, many medical school anatomy laboratories have difficulties maintaining adequate quantities of specimens in the needed conditions for educational study [10]. The practice of only studying surface anatomy falls short when one needs to understand complex anatomical details, such as vasculature, complexities of nervous innervations, associated congenital heart defects, and other intricate aspects [7,10]. It should also be noted that some students don’t have access to a dissection opportunity, and in others, such environments can be a source of stress and anxiety [11,12]. In this regard, the integration of XR technologies into medical education represents a significant advancement for many learners in the application of a better understanding of complex human anatomical and clinical knowledge required in healthcare or the medical device field.

Extended reality technologies are an umbrella term that encompasses VR, augmented reality (AR), and mixed reality (MR) educational tools [13,14]. In general, VR is typically described as a computer-generated three-dimensional space that simulates the presence of physical objects through multisensory interactions, creating immersive experiences in which users perceive artificial worlds as if they were real. It can also be characterized by its capacity to be a computer-generated space where multiple users can have simultaneous access and thus participate in shared activities [15-17]. While different types of metaverse share common features such as head-mounted displays for virtual immersions, motion tracking with 6-degree of freedom for real-time interactions, haptic feedbacks, and spatial audio to create a more immersive experience [15,18], VR differs from XR in that XR superimposes digital information onto the user’s real physical environment, while VR completely isolates the user from the outside world, immersing them in a fully simulated virtual environment with which they can interact [15,19].

According to recently reported studies on knowledge construction, perhaps a more effective way to better retain knowledge is through “active learning” or “learning by doing.” This implies that individuals better acquire critical knowledge through interactive and self-directed activities, such as those offered by VR [20]. The advantages of VR in educational settings include its ability to overcome physical barriers, allowing learners to engage with complex content regardless of location or access to physical resources [15]. This feature also enables collaboration among multiple users, providing greater flexibility to develop new applications and initiate diverse activities [6]. For example, by engaging with these VR applications, students can pursue self-directed learning and address their questions with greater autonomy [15]. Within the Visible Heart Laboratory, virtual platforms with interactive 3D models have been successfully used by medical students and residents for training relative to both cardiac physiology and transesophageal echocardiography [4,21]. By leveraging an extensive collection of over 650 perfusion-fixed human cardiac specimens, our laboratory continues to generate high-resolution 3D computational models that, in turn, facilitate precise virtual placements of medical devices [6]. Additionally, multiple applications have been developed and used in the field of presurgical planning, using VR in our facilities, and the unique availability of an extensive range of fixed human hearts. This approach has been the cornerstone for generating multiple educational and collaborative tools such as our Stenting simulator, Seldinger technique guidewire simulator, and augmented reality applications like the “Heart to Learn App” [22,23].

To evaluate the relative efficacies of using VR for human anatomic education, multiple research groups have consistently described the associated improvements this approach offers. For instance, Minouei et al [24] and Liu et al [25], in systematic reviews, found that VR commonly enhanced the students’ academic progress in terms of theoretical knowledge, practical proficiency, and overall satisfaction when used as a supplementary methods to other teaching approaches. Similarly, Baek et al [2] described 6 VR applications for the visualization and study of anatomical structures. They recommend that the use of these applications should be tailored to the student’s needs and suggested several ideal conditions for the creation of a VR application, such as the need for detailed tutorials, a realistic description of the anatomical model, motion animations, periodic updates, cost effectiveness, and high resolution. Other educators have conducted a controlled clinical study, examining the roles of interactive 3D models in medical anatomy education; in such, 200 medical students were surveyed, and the results showed a statistically significant improvement in post-test knowledge when compared to the control group [26]. Further, Garcia-Robles et al [27] and Salimi et al [28], in their meta-analyses, demonstrated that XR generated increases in knowledge compared to traditional learning methods, especially when used with complementary resources. They noted that these learning approaches were most beneficial for undergraduate students, of whom 80% reported that when they used such VR tools, they were useful for learning complex anatomies. Similarly, Liu and Wang et al [25] reported a higher satisfaction rating in groups of health care workers and students who used VR compared to other learning methods.

It is also important to consider the limitations of VR use in some individuals, which can generate adverse effects such as visually induced motion sickness (VIMS). In a pilot study, VIMS was reported in 32% of participants, of which 40% stated that experiencing VIMS could negatively impact their learning process when using VR educational tools [29].

One should also consider that gaps currently persist in the specific implementations of video game engines for applications in educational cardiac modeling, such as the implementation of haptic devices to simulate organ deformation by touching it, gamification strategies (rankings, scores), developing effective ways to reduce side effects (blurriness, dizziness), and performing more studies with a large sample size to assess the best way to deliver immersive education to students; this is particularly true in the field of CHD [30,31].

This study proposes an innovative pipeline that combines high-fidelity 3D modeling based on real tomographic data with dynamic visualization of myocardial layers and blood volume, and the implementations of such in Unity 3D [32], to create a VR-based application that can be used for educational purposes in CHDs, we want to measure acceptance and suitability of this application on medical practice by assessing possible future users of the application.

## Methods

### Image Acquisition and 3D Modeling

The methodologies employed in our work were developed in several sequential stages, beginning with the acquisition and processing of medical images. After informed consent, collection of primary and secondary medical data and information de-identification, anonymous DICOM images from patients were obtained through computed tomography (CT) from cases shared by Hospital La Cardio (Bogotá, Colombia), and from the anonymous database within the Visible Heart^®^ Laboratories (University of Minnesota, Minneapolis, USA). Following their initial clinical use, associated images were anonymized using the safe-harbor method to ensure patient confidentiality; the resulting dataset contained only the anonymized DICOM files required for 3D reconstruction. No additional consent was required during the development of this study, as the anonymized data posed no risks to patient privacy.

These image datasets were processed using Materialize Mimics Core 27.0 software to perform semi-automatic segmentation of cardiac structures. As these investigations and development of de-identified 3D computation models did not directly involve human participants or use identifiable personal data, that is, they did not require approval from an ethics committee.

In general, the segmentation process included automatic image alignments to ensure anatomical symmetry, followed by blood volume segmentations of ventricles, atria, and great vessels using adaptive thresholding (HU: approximately 200 to 1500) via the software’s CT Heart tools. For a given heart, specific cardiac defects were identified, such as interventricular communications, interatrial communications, patent ductus arteriosus, and/or others. Anatomical borders were manually refined using the Edit Masks function, achieving precision up to 1 mm, and internal hollowing was applied to simulate cardiac cavities.

Subsequently, these segmented congenital heart models were exported to Materialise 3-matic Medical 19.0 for optimization and final 3D modeling. At this stage, topological mesh repair was performed, eliminating nonmanifold triangles, and cardiac valves were added using a parametric model library, adjusting their scale to the specific dimensions of each patient’s heart. Anatomical textures were applied, and surfaces were smoothed using the Smooth Surface software module. The final models were exported in OBJ format, with mesh parameters optimized for VR performance.

### VR Application Development

During the development of the VR-based educational application, Unity software 2021.3 LTS with XR Interaction Toolkit 2.3 was used. The generated 3D heart models were then obtained from 3-matic and were imported to Unity as optimized mesh assets. To ensure complete visualization of the cardiac structures, backface culling was disabled, allowing the users to actively “cut” into the heart and analyze the internal structures of the given 3D heart model.

To optimize anatomical accuracy, custom shader-based materials were added with deoxygenated and oxygenated blood volumes color-coded with blue and red, respectively, as well as myocardium rendered in flesh tones, and the various pathological defects were highlighted: remaining visually coherent under a dynamic real-time clipping system, developed using shader programming.

A floating in-world Canvas menu was designed using Unity’s XR Ray Interactor system, allowing users to have seamless navigation between heart models, toggling different cardiopathies, and scrolling texts. Beyond single-user exploration, this unique educational application was designed to support real-time multiplayer interactions, using Unity’s Netcode for GameObjects: users can communicate in real time using integrated Vivox Voice Chat and Photon PUN networking.

While the initial release targets personal computer–based VR setups, a standalone version was optimized for lower-performance hardware using texture compression and GPU instancing to maintain rendering efficiency. Compatibility with OpenXR ensures broader accessibility across multiple headsets, including the Valve Index, HTC Vive, and Oculus Rift.

#### VR App: Innovative Functionalities

**Figure 1.**
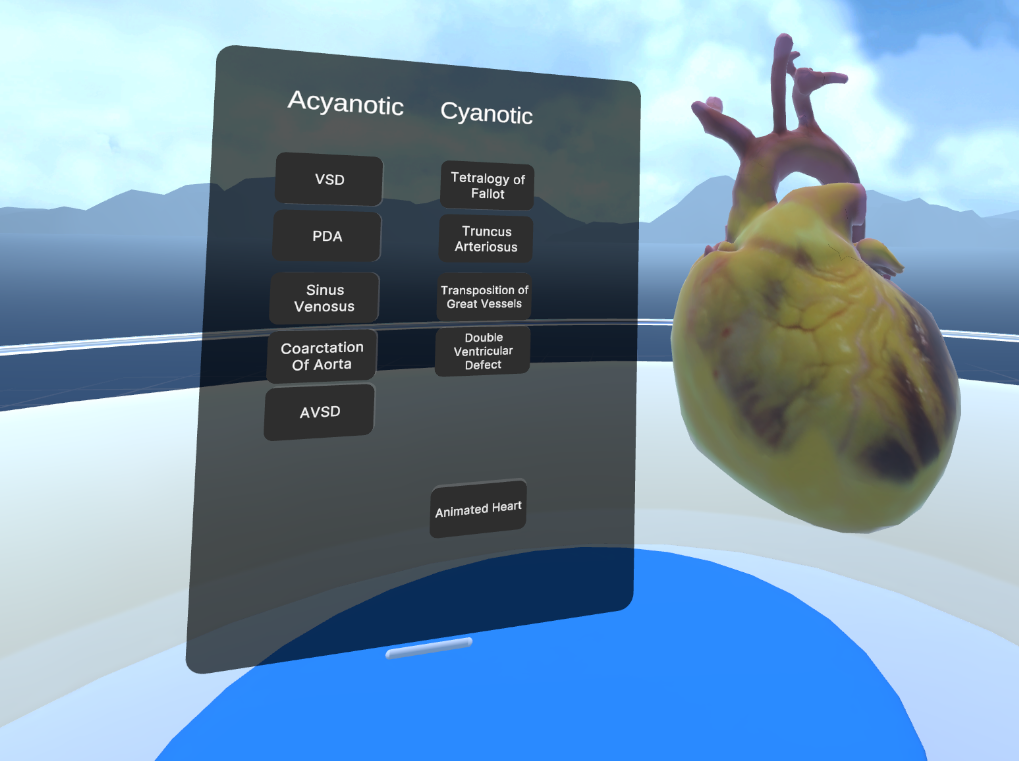
Congenital heart defects floating menu with animated normal heart.

**Figure 2.**
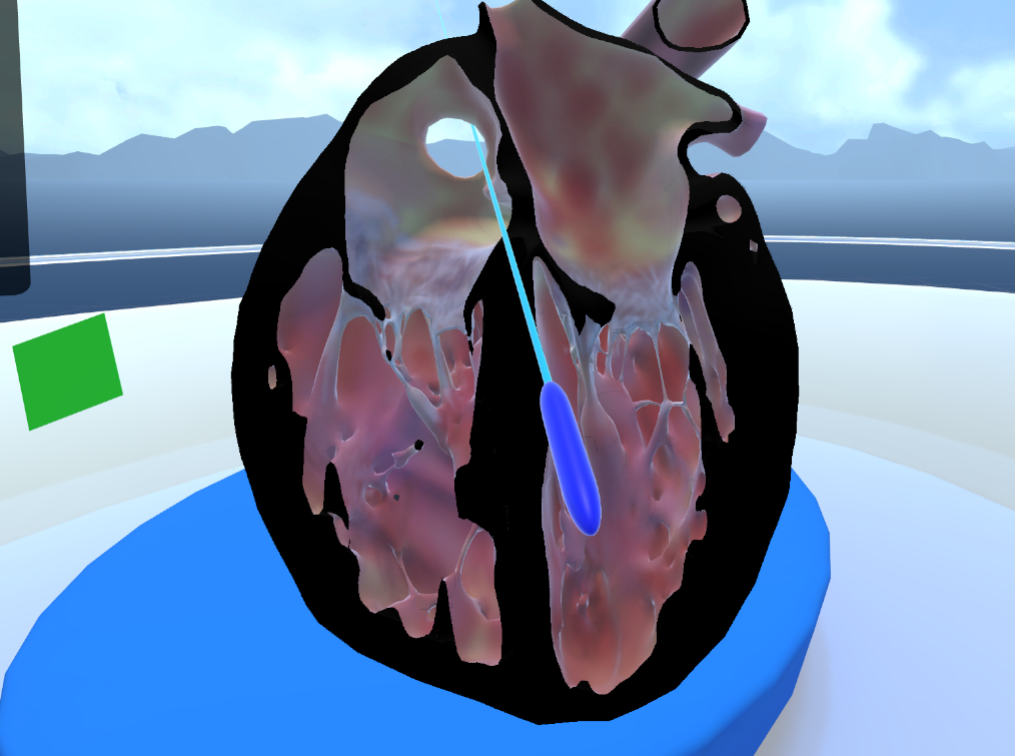
Pointer and cutting plane tools for this virtual reality application.

Our developed educational application includes a floating menu that categorizes heart defects into cyanotic and noncyanotic (Figure 1), with dynamic descriptive text incorporated within relative to each heart condition to be studied and explanatory images of predictive blood flows. Users can move the menu in the VR space as needed. The main functionalities allow users to manipulate cardiac models, including scaling, free rotations, and the ability to perform multiplanar cuts using Shader Graph. Additionally, there is an incorporated pointer feature that any of the users can use to mark the given defect to be mentioned or mark a specific location on the heart (Figure 2).

**Figure 3.**
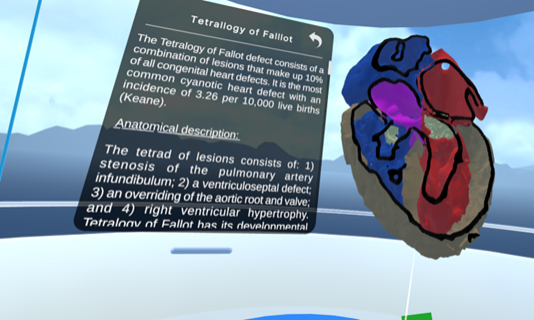
Tetralogy of Fallot heart model and description.

Multiple users can also freely interact with a heart with normal anatomy, which is animated to visualize how different structures of a normal heart, such as valves and myocardial walls, interact during the cardiac cycle of systole and diastole. Additionally, users can interact with various pathological models adapted from real tomographies, such as: for noncyanotic heart defects, a ventricular septal defect, a sinus venosus type atrial septal defect, a patent ductus arteriosus, an atrioventricular septal defect, a coarctation of the aorta, and for cyanotic heart defects, with truncus arteriosus, transposition of the great arteries, and tetralogy of Fallot (ToF) (Figure 3).

**Figure 4.**
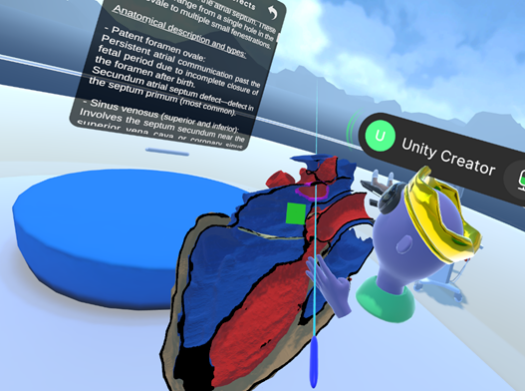
Collaborative multiplayer environment.

To facilitate collaborative teaching, a multiplayer architecture was implemented using Netcode for GameObjects, allowing synchronization of 3D models and actions across multiple devices in different global locations (Figure 4). This enables the use of a virtual classroom where the teacher has the abilities to instruct anatomy classes in real-time, point out defects with simultaneous visualizations by students, and guide anatomical tours, while students can access interactive menus and communicate with each other as well as the teacher for more immersive experiences.

### Participants and Intervention

To evaluate the functionalities of our newly developed application, this study utilized a cross-sectional observational design, it was conducted at the Visible Heart Laboratories (University of Minnesota, Minneapolis, USA). Data collection took place between 09/16/2025 to 09/20/2025.

The study used a convenience sampling method; a heterogeneous group of experts in cardiology, biomedical engineers, and medical trainees who visit our facilities for training on cardiology procedures and research purposes were invited to participate in our pilot study. Participants were provided with an Information Sheet for Research outlining the study procedures. Given that no Protected Health Information was being collected, the Information Sheet for Research provided before the data collection served as an Informed Consent process. Note, all participation was voluntary, and no incentives were given (Multimedia Appendix 1)*.*

Users were asked to try the VR tool in person after having received detailed instructions on how to use the educational tool. After that, it was required for the participants to select a given CHD, manipulate a VR heart, and find where the defect is located, slicing the heart with a virtual multiplanar cut, to identify the specific defect within the heart in different points of view. Participants were given the opportunity to freely interact with the VR environment for 15 minutes. After the VR intervention. Finally, after the intervention, participants completed a professional background and a 23-item training satisfaction survey. Survey responses were collected via an electronic form; these were filled out with a personal device. Data were collected anonymously using online Google Forms (Google LLC).

The sample size was determined by the availability of eligible participants visiting the laboratory during the data collection window. To minimize recall bias, the survey was administered immediately after the VR experience. Selection bias is acknowledged due to the convenience sampling of individuals already present in a specialized cardiac research center.

### Collection Instruments

A QR code was provided to answer the survey in which we gathered information related to *Professional background:* Professional role, years of experience in the field, experience with VR, experience with CHD education; and *VR-application satisfaction survey* that was assessed using a Likert survey adapted from the previously validated System Usability Scale [33] and a validated measure of VR user experience [34], also some other additional satisfaction questions about educational challenges on CHD education, VR application evaluation and implementation feasibility, also some open-ended questions were given for qualitative feedback. All responses were collected in an unidentifiable manner on a Google Forms spreadsheet (MultimediaAppendices 1^3^)*.*

### Statistical Analysis

Anonymized raw data were exported from Google Forms into Microsoft Excel for data organization. All statistical analysis was performed using Python (version 3.10) with Pandas and SciPy libraries. Descriptive statistics were conducted for all variables; categorical variables were reported as frequencies and percentages (n, %), whereas continuous variables were expressed as mean (SD) values, with 95% CI.

To determine the primary hypothesis regarding the application utility versus traditional methods effectiveness, a Wilcoxon signed-rank test was used to compare the responses within the same subjects (this test was selected due to sample size, N=9). One-sample Wilcoxon signed-rank tests were used to determine statistically significant responses different from neutral (score=3). Statistical significance was defined as a two-tailed *P* value <.05.

### Ethical Considerations

The protocol of this study follows ethical guidelines of the 1975 Declaration of Helsinki, ensuring compliance with international standards for the ethical use of human data in research and was reviewed and approved by the IRB #CEIC-0074‐2022 by Hospital LaCardio (Bogota, Colombia), and by the IRB #MOD00055981 (Atlas of Human Cardiac Anatomy: pediatric heart defects) at the University of Minnesota (Minneapolis, MN, USA). The medical images used in this study were originally obtained for clinical and educational purposes, with informed consent secured from patients at the time of imaging for educational and research uses. Following their initial clinical use, associated images were anonymized to ensure patient confidentiality. No additional consent was required during the development of this study, as the anonymized data posed no risks to patient privacy.

## Results

### Background Information

A total of 9 participants completed the survey, in which 4 (44%) were cardiologists, 2 (22%) were medical trainees, 1(11%) a biomedical engineer PhD student, 1 (11%) a biomedical engineer faculty member, and 1 (11%) a cardiac researcher. Respondents reported a wide spectrum of VR familiarity, from no prior experience to regular users, with 1 person having more than 20 years of experience, 1 user having between 11‐20 years of experience, 2 users between 6‐10 years of experience, 4 users having between 2‐5 years of experience and 1 person having less than 2 years of experience in their respective fields. Of those, 5 users described themselves as being “moderately familiar” with the use of VR, 1 user self-described as “very familiar,” and 3 users self-described as being “expert level” (See Table 1).

**Table 1.** Participants characteristics.

Characteristics and Category	Participants (N=9), n (%)
Professional role	
Pediatric cardiologist	3 (33.30)
Medical student/Resident	2 (22.20)
Adult CHD^a^ cardiologist	1 (11.10)
Cardiac researcher	1 (11.10)
Bioengineering faculty	1 (11.10)
BME PhD student	1 (11.10)
Years of experience	
2‐5 years	4 (44.40)
6‐10 years	2 (22.20)
<2 years	1 (11.10)
11‐20 years	1 (11.10)
>20 years	1 (11.10)
Prior VR^b^ experience	
Moderate (used several apps)	4 (44.40)
Extensive/Expert	2 (22.20)
Limited/None	3 (33.30)

a CHD: congenital heart defect.

b VR: virtual reality.

### Perceived Effectiveness: VR vs Traditional Methods

**Figure 5.**
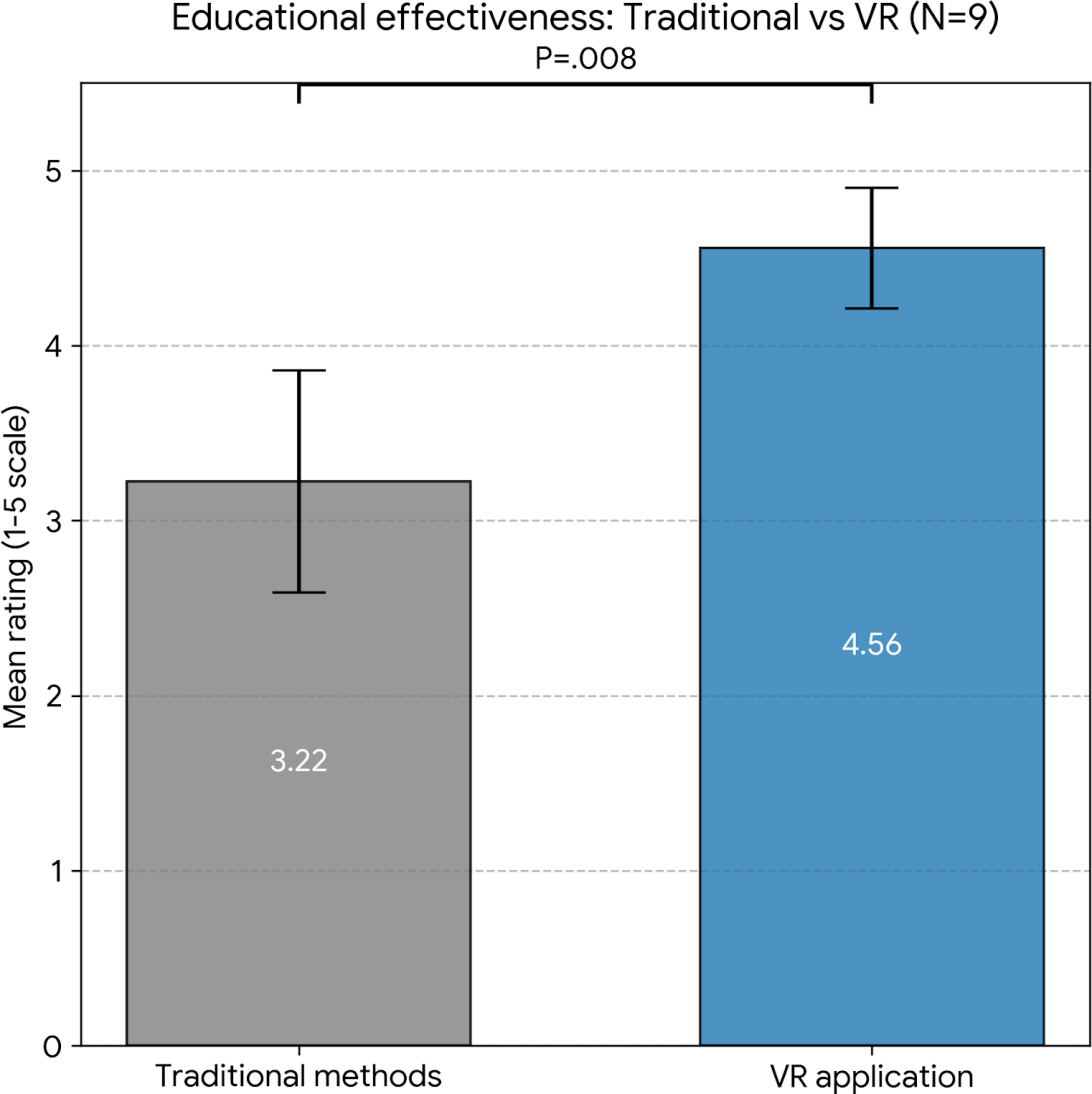
Perceived effectiveness of VR-based education versus traditional methods. VR: virtual reality.

Participants rated their perception of potential educational value for the VR app higher with a mean of 4.56 (SD 0.53), than traditional methods’ effectiveness with a mean of 3.22 (SD 0.97), with a significant statistical difference (*P*=.008), using a Wilcoxon signed rank-test (See Figure 5).

### Perceived Suitability in Different Educational Contexts

**Figure 6.**
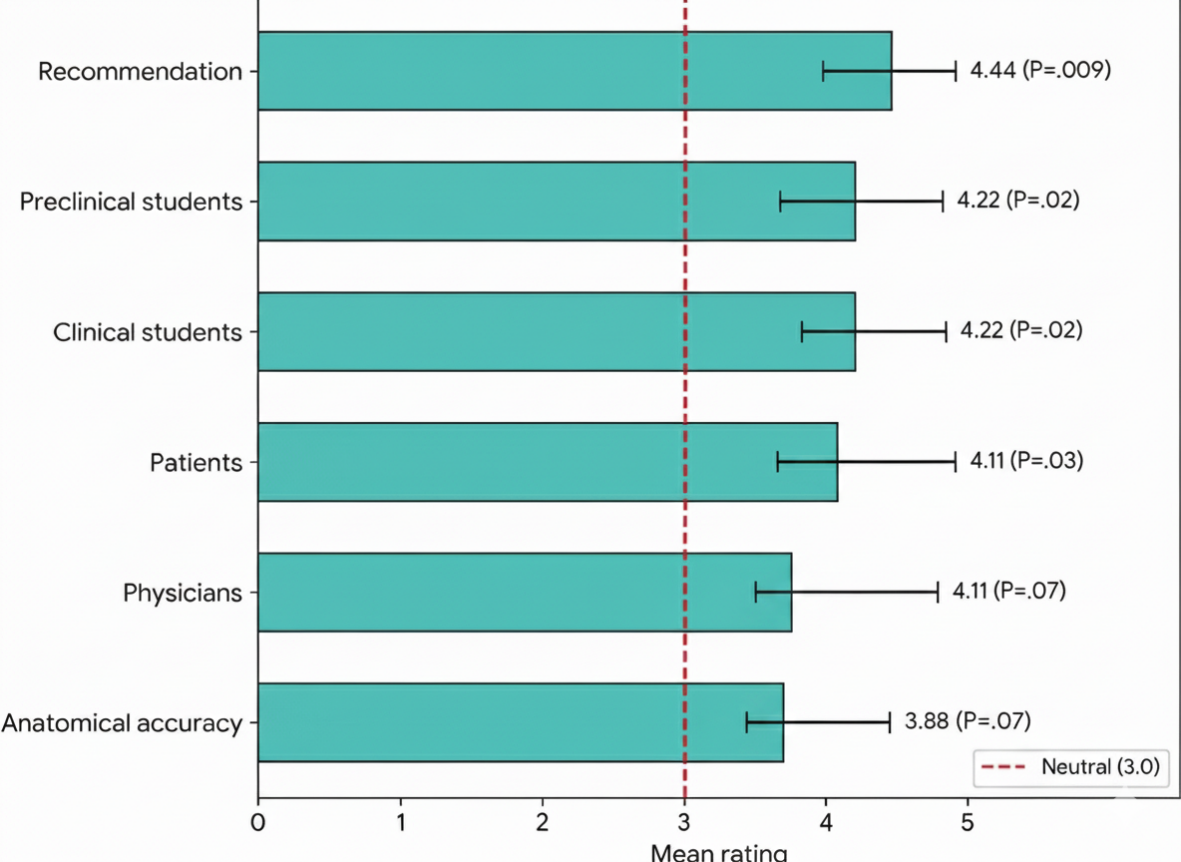
Perceived suitability for educational contexts.

Participants evaluated the application’s suitability for various target audiences given its educational content; they rated the application suitable for medical students at preclinical and clinical years, (mean 3.22, *P*=.02) and for patient education (mean 4.11, *P*=.03). Participants were likely to recommend the application (mean 4.44, SD=0.73, *P*=.009). Ratings for anatomical accuracy (mean 3.88) and suitability for practicing physicians (mean 4.11) were positive but not statistically significant. (See Table 2 and Figure 6).

**Table 2.** Perceived effectiveness and suitability of VR-based^a^ education for congenital heart defect.

Survey item	Mean (SD)	*P* value
Primary comparison		
Traditional methods effectiveness	3.22 (0.97)	Ref
VR application educational value	4.56 (0.53)	.008
Suitability		
Likelihood to recommend	4.44 (0.73)	.009
Suitability: Preclinical students	4.22 (0.83)	.012
Suitability: Clinical students	4.22 (0.83)	.012
Suitability: Patient education	4.11 (1.05)	.03
Suitability: Practicing physicians	4.11 (1.27)	.07
Anatomical accuracy	3.88 (1.13)	.07

a VR: virtual reality.

### Usability and Feasibility

It should be noted that the main significant challenges of CHD education, recognized by the users, were related to the complexities of the 3D spatial relationships recognized by 9 out of 9 (100%) of users, as well as the lack of interactive learning tools 6 (66%), and the limited availability of specimens and cases 3 (33%). The ongoing educational methods most commonly used by the users for CHD education were 2D illustrations/textbooks 9 (100%), followed by PowerPoint presentations 8 (88%) and clinical cases or rotations 4 (44%). Most users agreed that one of the most innovative features of our developed VR application was having real high-resolution CT scan-based 3D models 9 (100%) and real-time multiplanar cutting tool 7 (77%). Regarding the relative accuracies of the models, 3 (33%) of the respondents noted that they were found to be extremely accurate, 2 (22%) thought that they were very accurate, 2 (22%) noted that they were moderately accurate, and 1 (11%) considered them slightly accurate.

Regarding the feasibility of implementing these CHD learning tools, the most noted concerns for this VR application were technical complexity for users 6 (66%), 2 (22%) of individuals thought that the models needed to be more moderately accurate, and most felt that the costs of VR hardware 6 (66%) were a barrier. Also, 6 (66%) of users noted that one of the most critical barriers for ultimately implementing this VR tool at their institution would be related to budget constraints. When we asked the question, what additional features would enhance the educational value of this VR application, 6 (66%) of the users recommended adding post-surgical procedural simulations, while 5 (55%) recommended built-in assessment/quiz tools. Others noted that haptic feedback capabilities and additional cardiac pathologies would also be of value.

### Qualitative Feedback

For the obtained feedback on the open-ended questions, a large majority of users praised the CHD VR application’s ability to visualize complex 3D anatomy and their relationships with associated defects. Most respondents commonly mentioned that the benefit was improving anatomical understanding. Users noted:

It offers a lot better 3D understanding of the anatomy.

and

It can help better visualize 3D interactions and gather people without geographical constraints.

Yet it should be noted that the participants most commonly recommended adding blood-flow simulations and improving user interfaces for collaboration, which would help guarantee that the user is able to follow the educational purpose of the app. One suggested:

Have better CHD models, be more intuitive, and have a set of cut hearts already. Also, adding blood flow through the defects would be very interesting.

Others advised about the risks of losing face-to-face interactions:

Needs to guarantee that the student/patient is able to follow, we are losing the face-to-face interaction, and we would not be able to tell if they are following, listening, understanding, etc.

Most of the common concerns about implementing such VR technology in the educational field were related to technical support, hardware costs, and continued maintenance, as well as educational institutions’ resources. Users displayed reservations about:

Widespread implementation across places with different financial resources.

The maintenance of VR systems and the longevity of the technology.

Most respondents agreed that our CHD VR tool would be most effective in small group sessions, in self-directed learning, or within one-on-one patient interactions.

## Discussion

### Principal Findings

Our findings demonstrate that a VR application integrated with specific CHD models from real CT images is perceived of high educational value for CHD education, compared to traditional methods by a group of experts and trainees, consistent with previous literature [25,31]. While other similar applications exist [30,31], participants highlighted the integration of CT scans to generate a high fidelity heart model of great value (9 out of 9 participants, 100%), as well as the ability to perform multiplanar real-time sectioning on the heart models serving as a virtual dissecting platform to overcome physical constrains, such as, limited availability of specimens, noted by 3 out of 9 participants (33%).

### Educational utility

Most survey respondents also agreed that small group sessions and an independent learning setting could be the main uses for this application, specifically for medical education training and patient education, which is consistent with previous systematic reviews and meta-analyses on XR and medical education on knowledge improvement [27,28]. Of interest, most suggested future features we should consider for future implementations should include: post-surgical procedure simulations, additional cardiac pathologies, progress tracking and quiz tools, multi-language support, as well as AI integration.

Thus, our work should contribute to the field of digital medical education by providing a reproducible framework for developing immersive cardiac simulators, while simultaneously addressing technical and pedagogical aspects. Note that, unlike commercial VR solutions, our approaches prioritize didactic customizations, facilitating curricular adaptations. Developed educational VR applications in general offer multiple advantages over traditional learning methods. Here, we discuss the ability to allow multiplayer visualization, real-time interactivity with complex cardiac structures, active and collective learning in a virtual classroom, and increased accessibility. These features require the use of VR, a valuable supplement to traditional education methods, potentially helping to reduce stress and anxiety for some students [10,27]; but some may induce undesired side effects.

### Limitations

Despite its potential learning advantages, it is important to consider some limitations of VR use, which can cause adverse effects such as visually induced motion sickness or “cybersickness” that may negatively impact or even limit the uses of these learning experiences for some users [29,35]. Similar to what was described in other VR educational studies, even though users think that this is a good adjunctive tool that could be used for medical education, they also believe that potential drawbacks for this could be software and hardware maintenance issues. Additionally, the technological learning curves can vary from user to user, depending on their initial familiarity with controls and video game usage. Further, for some, implementation costs can also be a factor, including expenses associated with hardware, software, and human resources such as systems engineers, computer scientists, graphic designers, healthcare personnel, and researchers, among others [6]. We acknowledge that a potential novelty bias associated with VR educational technologies should be acknowledged by lessening the educational outcomes of subjects interacting with new technologies for the first time [36]. The use of convenience sampling with the participants of different activities at a cardiovascular laboratory means that participants will have higher knowledge and/or interest in cardiovascular VR applications, which poses one of the greatest limitations of this study. It is important to note that a small sample size limits the statistical power and generalizability of the study.

### Future directions

Furthermore, long-term comparative controlled clinical studies should be designed and performed associated with our developed VR platform, to assess knowledge retention in students and/or patients with congenital heart diseases. It is also important to consider other ethical implications of using VR of real patient anatomy in medical education. Additionally, the costs of such technologies could, in part, create educational inequalities in regions with fewer economic resources. Finally, it is essential to ensure that students do not develop an excessive dependence on VR, neglecting other essential practical skills such as direct tissue manipulations and/or dissections.

### Conclusions

This framework portrays the integration of an innovative VR application with high-fidelity CT scans of Congenital Heart Defects as a potential tool for medical and patient education. Our findings suggest that VR could be easily implemented and adapted by medical educators to bridge the cognitive gap in understanding complex defects. This work also serves as a stepping stone into a more advanced and technological approach for education using newer tools like AI or XR as educational tools for medical curricula. Furthermore, its ongoing development and continuous collaboration between multiple research centers and academic institutions could have a substantial impact on the training of future healthcare professionals and on patient education. The VR platform we described here could help all types of learners better understand CHDs, provide a means for clinicians around the world to discuss complex CHD and treatment approaches, and aid medical device designers relative to future innovations.

## Supplementary material

10.2196/74429 Multimedia Appendix 1 Information sheet.

10.2196/74429 Multimedia Appendix 2 Implementation survey.

10.2196/74429 Multimedia Appendix 3 Virtual Reality app implementation survey (Likert Style Table).

## Abbreviations

AR: augmented reality
CHD: congenital heart defects
CHD: congenital heart defects
CT: computed tomography
GAI: generative artificial intelligence
VIMS: visually induced motion sickness
VR: virtual reality
XR: extended reality
