# Supplementary material for: Virtual Reality Application for Teaching Complex Congenital Heart Defect Anatomy: Design and Development Study
Source: JMIR XR Spat Comput. 2025 Dec 22;2:e74429. doi: 10.2196/74429 (PMC13202504; doi:10.2196/74429)
Supplement: Multimedia Appendix 1 [file xr-v2-e74429-s001.docx]

**Appendix 1. Information Sheet**

INFORMATION SHEET FOR RESEARCH

**Design and development of an Innovative Virtual Reality Application for Teaching the Anatomy of Various Complex Congenital Heart Defects**

You are invited to be in a research study of the implementation of Virtual Reality as a potential adjuvant tool for education in congenital heart defects. You were selected as a possible participant because you are part of a heterogeneous group of stakeholders and/or potential users for this innovative tool. We ask that you read this form and ask any questions you may have before agreeing to be in the study.

**Activities:**

If you agree to be in this study, we would ask you to do the following things:

 -We will explain you how to use a VR headset and controllers, a 5 minute instructional demonstration will be given to understand how to use this application, and you will be given 15-20 minutes to freely interact with the the application, with the objective of being able to move around the VR world, interact with the objects and locate one congenital heart defect within the VR heart models.

-After this intervention you will be asked to complete this survey. Estimated time to complete: 15-20 minutes

-This survey is designed to gather expert feedback on our innovative Virtual Reality (VR) application for teaching congenital heart defects (CHDs). Your expertise is valuable in helping us validate the design, assess feasibility, and identify areas for improvement before broader implementation.

**Confidentiality:**

All responses will remain confidential and will be used solely for research purposes to improve our VR educational tool.

During the project, information from this study will be kept private and will be stored securely. Only the research team will have access to information that identifies you. Your identifying information will not be shared with others outside of this research study. However, organizations that may inspect and copy your information include the Institutional Review Board (IRB), the committee that provides ethical and regulatory oversight of research, and other representatives of this institution, including those that have responsibilities for monitoring or ensuring compliance (such as the Quality Assurance Program of the Human Research Protection Program (HRPP)).

Any personal information that could identify you will be removed or changed before we publish any report or share the results or data from this study.

**Voluntary Nature of the Study:**

 Participation in this study is voluntary. Your decision whether or not to participate will not affect your current or future relations with the University of Minnesota

**Will I be compensated for my participation?**No compensation will be given after participating in this survey.

**To Contact the Research Team:**

 The researcher(s) conducting this study is (are): Paul Iaizzo and Kevin Muneton. You may ask any questions you have now. If you have questions later regarding the study or your participation, you are encouraged to contact the research team at University of Minnesota, phone number: 5618713359, kmuneton@umn.edu.

**To Contact Someone Outside of the Research Team:**

This research has been reviewed and determined exempt by the Institutional Review Board (IRB), which is part of the Human Research Protections Program (HRPP). If there is an issue you would like to discuss with someone who is not on the research team you are encouraged to call the HRPP Research Participants’ Advocate Line at 612-625-1650 (Toll Free: 1-888-224-8636) or go to[z.umn.edu/participants](https://research.umn.edu/units/hrpp/research-participants/questions-concerns). For example:

●       Your questions, concerns, or complaints are not being answered by the research team.

●       You are having difficulty reaching the research team.

●       You want to talk to someone besides the research team.

●       You have questions about your rights as a research participant.

●       You want to provide feedback about this research to someone who is not on the study team.
