# Supplementary material for: Virtual Reality Application for Teaching Complex Congenital Heart Defect Anatomy: Design and Development Study
Source: JMIR XR Spat Comput. 2025 Dec 22;2:e74429. doi: 10.2196/74429 (PMC13202504; doi:10.2196/74429)
Supplement: Multimedia Appendix 2 [file xr-v2-e74429-s002.docx]

**Appendix 2. Implementation Survey**

***I. Professional background information***

1. What is your primary professional role?

[ ] Medical educator/faculty

[ ] Pediatric cardiologist

[ ] Cardiac surgeon

[ ] Medical student/resident

[ ] Curriculum developer

[ ] Educational technology specialist

[ ] Other

2. How many years of experience do you have in your field?

[ ] Less than 2 years

[ ] 2-5 years

[ ] 6-10 years

[ ] 11-20 years

[ ] More than 20 years

3. What is your experience with Virtual Reality technology?

[ ] No experience

[ ] Limited experience (tried once or twice)

[ ] Moderate experience (used several VR applications)

[ ] Extensive experience (regular user)

[ ] Expert level (develop or research VR applications)

4. How familiar are you with congenital heart defect education?

[ ] Not familiar

[ ] Somewhat familiar

[ ] Moderately familiar

[ ] Very familiar

[ ] Expert level

***II. Educational Need Assessment***

5. In your opinion, what are the most significant challenges in teaching congenital heart defects? (Select all that apply)

[ ] Limited availability of physical specimens

[ ] Complexity of 3D spatial relationships

[ ] Student anxiety around cadaveric material

[ ] Difficulty visualizing dynamic cardiac function

[ ] Limited access to diverse pathological cases

[ ] Lack of interactive learning tools

[ ] Time constraints in curriculum

[ ] Cost of educational materials

[ ] Other

6. How would you rate the current effectiveness of traditional methods for teaching CHDs? (1 = Not effective, 5 = Very effective) *(See Appendix 3)*

7. What traditional teaching methods do you currently use for CHD education? (Select all that apply)

[ ] Cadaveric specimens

[ ] Anatomical models

[ ] 2D illustrations/textbooks

[ ] Video recordings

[ ] PowerPoint presentations

[ ] Case-based discussions

[ ] Clinical rotations

[ ] None of the above

[ ] Other

***III. VR Application Evaluation***

8. How would you rate the potential educational value of this VR application? (1 = Not valuable, 5 = Extremely valuable) *(See Appendix 3)*

9. Which features of the VR application do you find most innovative? (Select up to 3)

[ ] Real CT scan-based 3D models

[ ] Multiplanar cutting/sectioning tools

[ ] Collaborative multiplayer environment

[ ] Interactive pointer system

[ ] Categorized cyanotic/acyanotic defects

[ ] Cross-platform compatibility

[ ] Real-time global collaboration

[ ] Color-coded blood flow visualization

10. How accurate do you consider the anatomical representation in the VR models?

[ ] Extremely accurate

[ ] Very accurate

[ ] Moderately accurate

[ ] Slightly accurate

[ ] Cannot determine from provided information

11 - 14. Suitability Ratings: How suitable would this VR application be for different educational levels? *(See Appendix 3)*

11, Medical students (pre-clinical): (Rate each on a 5-point scale: 1=Not suitable, 5=Extremely suitable)

12. Medical students (clinical years): (Rate each on a 5-point scale: 1=Not suitable, 5=Extremely suitable)

13. Practicing physicians: (Rate each on a 5-point scale: 1=Not suitable, 5=Extremely suitable)

14. Patient education: (Rate each on a 5-point scale: 1=Not suitable, 5=Extremely suitable)

15. What concerns do you have about implementing this VR application? (Select all that apply)

[ ] Technical complexity for users

[ ] Cost of VR hardware

[ ] Motion sickness/cybersickness

[ ] Learning curve for educators

[ ] Integration with existing curriculum

[ ] Maintenance and technical support

[ ] Student overreliance on technology

[ ] Reduced hands-on learning opportunities

[ ] Data privacy concerns

[ ] No significant concerns

[ ] Other

***IV. Implementation Feasibility***

16. How likely would you be to recommend this VR application to colleagues? (1 = Not likely, 5 = Extremely likely) *(See Appendix 3)*

17. What would be the most significant barriers to implementing this technology in your institution? (Choose 3)

[ ] Budget/funding constraints

[ ] Lack of technical infrastructure

[ ] Faculty training requirements

[ ] Student acceptance/adoption

[ ] Integration with current curriculum

[ ] Administrative approval processes

[ ] Maintenance and support costs

[ ] Space/facility requirements

[ ] Other

18. What additional features would enhance the educational value of this VR application? (Select all that apply)

[ ] Built-in assessment/quiz tools

[ ] Progress tracking analytics

[ ] Haptic feedback capabilities

[ ] Integration with Learning Management Systems

[ ] Additional cardiac pathologies

[ ] Surgical procedure simulations

[ ] Multi-language support

[ ] AI-powered personalized feedback

[ ] Other

***V. Feedback: Open-Ended Questions***

19. What specific advantages do you see in using VR technology for CHD education compared to traditional methods?

20. What are your main concerns or reservations about using VR for medical education?

21. How could this VR application be improved to better meet the needs of educators and students?

22. In what educational contexts do you think this VR application would be most effective? (e.g., classroom lectures, small group sessions, self-directed learning, etc.)

23. Any additional comments or suggestions regarding this VR application for CHD education?
